# Serum lipocalin-2 levels positively correlate with coronary artery disease and metabolic syndrome

**DOI:** 10.1186/1475-2840-12-176

**Published:** 2013-12-21

**Authors:** Jie Ni, Xiaojing Ma, Mi Zhou, Xiaoping Pan, Junling Tang, Yaping Hao, Zhigang Lu, Meifang Gao, Yuqian Bao, Weiping Jia

**Affiliations:** 1 Department of Endocrinology and Metabolism, Shanghai Jiao Tong University Affiliated Sixth People’s Hospital, Shanghai Clinical Center for Diabetes, Shanghai Key Laboratory of Diabetes Mellitus, Shanghai Diabetes Institute, 600 Yishan Road, Shanghai, 200233, China; 2 Department of Cardiology, Shanghai Jiao Tong University Affiliated Sixth People’s Hospital, Shanghai, China

**Keywords:** Atherosclerosis, Coronary artery disease, Metabolic syndrome, Serum LCN2 levels

## Abstract

**Background:**

The lipocalin-2 (LCN2) cytokine, primarily known as a protein of the granules of human neutrophils, has been recently reported to be implicated in metabolic and inflammatory disorders. This study was designed to evaluate the relationship between serum LCN2 levels and coronary artery disease (CAD).

**Methods:**

Serum LCN2 levels of 261 in-patients who underwent coronary angiography were measured by sandwich enzyme immunoassay. Demographic (169 men and 92 postmenopausal women) and clinical (metabolic syndrome (MS), triglyceride (TG) and C-reactive protein (CRP) levels) characteristics were collected to assess independent factors of CAD (CAD: 188 and non-CAD: 73) and serum LCN2 levels by multiple logistic regression and multivariate stepwise regression analyses, respectively.

**Results:**

Serum LCN2 levels were significantly higher in men (37.5 (27.4-55.4) vs. women: 28.2 (18.7-45.9) ng/mL, *p* < 0.01) and men with CAD (39.2 (29.3-56.5) vs. non-CAD men: 32.7 (20.5-49.7) ng/mL, *p* < 0.05), and showed significant positive correlation with CAD in men (odds ratio = 2.218, 95% confidence interval: 1.017-4.839). Similarly, serum LCN2 levels were significantly higher in men with MS (40.2 (31.9-59.4) vs. non-MS: 32.0 (21.7-47.6) ng/mL, *p* < 0.01) and showed a significant positive correlation with the number of MS components (*p* for trend < 0.05). No significant differences or correlations were seen in women. TG and neutrophils (standard β = 0.238 and 0.173) were independent factors of serum LCN2 levels in men, and only neutrophils (standard β = 0.286) affected levels in women (all *p* < 0.05).

**Conclusions:**

Increased serum LCN2 levels are positively correlated with the presence of CAD and MS in a Chinese cohort.

## Introduction

Coronary artery disease (CAD) is commonly encountered in long-term and urgent clinical care settings, yet high mortality and disability rates persist [1]. The fundamental pathological change observed in CAD patients is atherosclerosis, and this cholesterol and lipid-based blockage has also been implicated in metabolic disorders and chronic inflammation [2,3].

The extensive research efforts put forth to elucidate the molecular mechanisms of CAD pathogenesis have indicated a potential role for the adipocyte-related proinflammatory cytokine lipocalin-2 (LCN2). It has also been detected in some organs with normal metabolism, including bronchus, stomach, small intestine, pancreas, kidney, prostate gland and thymus. Additionally, LCN2 can be secreted by activated neutrophils, adipocytes and macrophages [4]. While in metabolic and inflammatory disorders, high LCN2 levels were noticed in adipocytes [5]. Therefore, LCN2 has been characterized as a potential contributing factor to obesity, insulin resistance, hyperglycemia, chronic inflammation [5,6], apoptosis, and impaired renal function [7-9].

LCN2 exerts structural effects which can perturb normal physiological functions. For example, LCN2 complexes with matrix metalloproteinase-9 (MMP-9) and contributes to fibrosis, and this functional interaction is recognized by the alternative designation of LCN2 as the neutrophil-gelatinase associated lipocalin (NGAL) [10]. In addition, Lcn2 expression has been detected in clinical specimens of atherosclerosis plaques (particularly in the vascular endothelial cell, smooth muscle cell and macrophage components) and damaged myocardium, as well as in vitro analysis of arterial plaques [11,12]. Studies in animal models have shown that murine atherosclerosis is accompanied by increased levels of serum LCN2 and that conditions of hypoxia and myocardial infarction (MI) induce Lcn2 mRNA expression [13]. Serum LCN2 levels have also been found to be related to glucose metabolism and blood lipid composition [14,15].

In recent years, the possible association of serum LCN2 levels and CAD has been addressed by clinical trials, and studies based on Caucasian and Korean populations have suggested positive associations between expression of this multifunctional protein and development of this life-threatening disease with systemic implications [16-18]. However, no study to date has assessed the relationship between serum LCN2 levels and CAD in a Chinese population. The current study was designed to investigate the serum LCN2 levels detected in Chinese patients who underwent coronary angiography (CAG) to address symptoms of chest pain or/and chest tightness and determine the independent factors of CAD and increased serum LCN2 levels.

## Materials and methods

### Patient selection and demographic/clinical characteristics

Between July 2008 to January 2010, the in-patient population of the Department of Cardiology of Shanghai Jiao Tong University Affiliated to the Sixth People’s Hospital was searched for patients who were admitted to undergo CAG to address reported experience of or current suffering from chest pain or/and chest tightness.

The identified patients were required to complete a standardized questionnaire upon enrollment to collect data on past and present illnesses, drug use, and smoking habits. Subjects were identified as ‘current smokers’ if they reported regular use of inhaled tobacco products or having smoked at least one cigarette per day at any time over the previous 6 months [19]. Patients were denied study enrollment if they reported MI occurrence at any time during the past 3 months, history of congestive heart failure (New York Heart Association Class III-IV) or related treatment (coronary bypass surgery or percutaneous coronary intervention) at any time during the past 6 months, ongoing hepatic or renal dysfunction, current acute infection, history of malignancy or autoimmune diseases, or diagnosis of mental illness.

The study was carried out with pre-approval from the local Ethics Committee of Shanghai Jiao Tong University affiliated Sixth People’s Hospital and complied with the Declaration of Helsinki. All enrolled subjects provided informed consent.

### Anthropometric measurements

A total of 261 patients were enrolled in the study, including 169 men and 92 postmenopausal women ranging in age from 39 to 86 years-old (mean age: 65.9 ± 9.5 years). A complete physical examination was given to each study participant upon enrollment, and included body mass index (BMI, calculated as kg weight/m^2^ height), waist circumference (W, measured at the midpoint between the lowest rib and the superior border of the iliac crest on the midaxillary line), and blood pressure (measured by conventional sleeve mercury sphygmomanometer).

### Laboratory measurements

Blood samples were taken from all study participants after an overnight fast (of ≥ 10 h) and stored at -80°C until testing. Fasting plasma glucose (FPG) and 2 hours postprandial glucose (2hPG) were determined by the glucose oxidase method. Fasting insulin was assayed by radioimmunoassay (Linco Research, St Charles, Missouri, USA). Insulin resistance was estimated via the homeostasis model assessment index (HOMA-IR) [20]. Glycated hemoglobin (HbA1c) concentration was measured by high-pressure liquid chromatography (Bio-Rad Inc., Hercules, CA, USA). Serum creatinine (Scr), uric acid (UA) and lipid profiles, including triglyceride (TG), total cholesterol (TC), high-density lipoprotein cholesterol (HDL-c) and low-density lipoprotein cholesterol (LDL-c), were determined by standard enzymatic procedures on an automated bioanalyzer (7600–020; Hitachi, Tokyo, Japan). The 24 h urine albumin (24hALB) concentration was measured by the standard rate nephelometry method. Estimated glomerular filtration rate (eGFR, mL/min/1.73 m^2^) was calculated according to the equation from the Modification of Diet in Renal Disease (MDRD) study: [186 × (Scr/88.4) ^-1.154^ × (age) ^-0.203^ × 0.742 (if women)] [21]. Serum C-reactive protein (CRP) was measured by particle-enhanced immunonephelometry assay (Dade Behring Inc., Newark, NJ, USA). Serum LCN2 and adiponectin levels were measured by sandwich enzyme immunoassays (Antibody and Immunoassay Services, The University of Hong Kong). Concerning the assay of LCN2, the immunoplate was pre-coated with a monoclonal antibody specific for human LCN2. A second horseradish peroxidase (HRP)-linked monoclonal antibody specific to human LCN2 was co-incubated with the samples. While for adiponectin, a mouse monoclonal antibody specific to human adiponectin was pre-coated onto a micro-tire plate and a second HRP-linked monoclonal antibody specific to human adiponectin was co-incubated with the samples. The inter- and intra-assay coefficients of variation were 6.77% and 1.84% for LCN2 and 8.6% and 7.3% for serum adiponectin.

### Diagnosis of metabolic syndrome

Metabolic syndrome (MS) was assessed among the study population according to the criteria published by the 2007 Chinese Joint Committee for Developing Chinese Guidelines on Prevention and Treatment of Dyslipidemia in Adults. Three of the following five components were required for MS diagnosis: 1) central obesity (W >90 cm for men and >85 cm for women); 2) fasting TG of ≥1.7 mmol/L; 3) fasting HDL-c of <1.04 mmol/L; 4) systolic blood pressure (SBP) of ≥130 mmHg and/or diastolic blood pressure (DBP) of ≥85 mmHg, or known treatment for hypertension; 5) FPG of ≥6.1 mmol/L and/or 2hPG of ≥7.8 mmol/L, or receipt of hypoglycemic therapy for diabetes [22].

### CAG and coronary stenosis index

Selective CAG was carried out with standard Judkins techniques [23] and all major coronary arteries were imaged in more than two orthogonal views. The analysis of angiographic images was carried out by two experienced cardiologists, who were blinded to the patients’ clinical information. CAD was diagnosed when ≥50% diameter lumen stenosis was detected in a major coronary artery, including the left main coronary artery, left anterior descending artery or its first diagonal branch, left circumflex artery or its first obtuse marginal branch, and right coronary artery. The severity of CAD was assessed by calculating the coronary stenosis index (CSI) as the sum of the following scores of stenosis for each lesion: none = 0; <25% = 1; 25-49% = 2; 50-74% = 3; 75-100% = 4 [24].

### Statistical analysis

All statistical analyses were carried out with the SPSS statistical software (version 16.0; Chicago, IL, USA). Variables with normal distribution are presented as mean ± standard deviation and variables with skewed distribution are expressed as median with inter-quartile range. Student’s *t*-test (for normally distributed variables) and Wilcoxon rank-sum test (for skewed variables) were used to assess the significance of differences found between the CAD group and the non-CAD group. Chi-squared test was used to assess between-group differences for dichotomous or categorical variables. Kruskal-Wallis rank-sum test was used to compare serum LCN2 levels in groups according to the number of MS components, and Spearman’s correlation was used to evaluate the relation between serum LCN2 levels and other clinical parameters. Multiple logistic regression analysis was performed to identify factors that were independently correlated with CAD, and multivariate stepwise regression analysis was used to further assess the independent correlated clinical parameters of serum LCN2 levels. The skewed variables had been transformed by natural logarithm before regression analyses. The threshold for statistical significance was a two-sided *p*-value of <0.05.

## Results

### Clinical characteristics of study participants

The serum LCN2 levels measured in the entire study population ranged from 5.8 to 221.1 ng/mL, with an average level of 34.9 (23.4-51.2) ng/mL. The average level in men was significantly higher than that in women (37.5 (27.4-55.4) vs. 28.2 (18.7-45.9) ng/mL, *p* < 0.01).

Compared with non-CAD subjects, the patients with CAD showed significantly higher age, CSI, and proportion of lipid-lowering therapy. Stratification analysis by gender showed that men with CAD had significantly lower uric acid levels than those without CAD and that women with CAD had significantly lower BMI but significantly higher proportion of hypoglycemic therapy than their non-CAD counterparts (all *p* < 0.05, Table 1).

**Table 1:** Characteristics of study participants according to the presence or absence of CAD

**Variables**	**Non-CAD**			**CAD**		
	**Total**	**Men**	**Women**	**Total**	**Men**	**Women**
N	73	38	35	188	131	57
Age (years)	63.6 ± 9.4	62.1 ± 9.7	65.2 ± 8.8	66.8 ± 9.5^a^	65.7 ± 9.9^b^	69.3 ± 8.0^c^
CSI	0 (0–1)	0 (0–1)	0 (0–1)	9 (5–14)^aa^	10 (5–14)^bb^	8 (4–14)^cc^
BMI (kg/m^2^)	25.5 ± 3.7	25.4 ± 4.1	25.6 ± 3.4	24.4 ± 3.1^a^	24.7 ± 3.1	23.9 ± 3.0^c^
W (cm)	90.7 ± 10.8	92.7 ± 11.9	88.5 ± 9.0	90.3 ± 8.8	91.7 ± 8.5	87.3 ± 8.7
SBP (mmHg)	130 (120–150)	130 (120–150)	130 (120–145)	133 (120–150)	130 (120–147)	140 (128–150)
DBP (mmHg)	80 (70–87)	80 (70–88)	80 (70–86)	80 (70–84)	80 (70–84)	75 (70–82)
FPG (mmol/L)	5.38 (5.07-6.17)	5.64 (5.12-6.23)	5.31 (5.00-5.96)	5.46 (5.04-6.57)	5.44 (5.04-6.41)	5.59 (4.99-7.64)
2hPG (mmol/L)	8.17 (6.27-9.97)	9.12 (6.79-10.55)	7.17 (5.27-9.29)	8.76 (6.66-11.92)	8.77 (6.71-12.16)	8.67 (6.49-11.72)
HbA1c (%)	6.2 (5.7-6.5)	6.2 (5.6-6.5)	6.2 (5.7-6.4)	6.2 (5.8-6.9)	6.2 (5.8-6.7)	6.4 (5.8-7.2)
HOMA-IR	4.1 (2.9-5.9)	3.9 (3.1-5.4)	4.9 (2.8-6.0)	4.0 (2.8-5.9)	4.0 (2.7-5.6)	4.3 (2.9-6.8)
TC (mmol/L)	4.53 ± 1.03	4.20 ± 0.95	4.90 ± 0.99	4.31 ± 1.10	4.17 ± 1.02	4.63 ± 1.20
TG (mmol/L)	1.63 (1.04-2.30)	1.62 (1.09-2.18)	1.66 (0.88-2.44)	1.51 (1.08-2.25)	1.49 (1.04-2.14)	1.57 (1.17-2.57)
HDL-c (mmol/L)	1.07 (0.88-1.37)	0.97 (0.81-1.38)	1.26 (1.06-1.37)	1.02 (0.87-1.28)	0.96 (0.85-1.16)	1.19 (0.93-1.35)
LDL-c (mmol/L)	3.06 ± 0.86	2.85 ± 0.90	3.30 ± 0.76	2.90 ± 0.99	2.83 ± 0.93	3.08 ± 1.10
Serum adiponectin (ug/mL)	7.8 (5.7-11.5)	7.4 (5.0-10.5)	8.9 (5.8-14.2)	7.0 (4.8-11.1)	6.5 (4.4-10.6)	7.9 (6.1-13.0)
UA (μmol/L)	341.6 ± 85.2	383.0 ± 79.5	295.2 ± 66.1	342.0 ± 82.7	349.1 ± 78.1^b^	325.7 ± 91.0
Scr (μmol/L)	73.2 ± 18.4	83.2 ± 15.8	61.9 ± 14.2	78.0 ± 19.4	83.8 ± 17.1	64.5 ± 17.7
24hALB (mg/d)	7.8 (4.9-17.1)	8.5 (5.6-18.1)	6.6 (3.4-15.3)	7.0 (4.7-14.3)	7.1 (4.6-12.9)	6.7 (5.1-19.6)
eGFR (mL/min/1.73 m^2^)	92.6 ± 22.7	90.3 ± 19.7	95.2 ± 25.7	89.6 ± 22.3	89.2 ± 21.3	90.7 ± 24.9
CRP (mg/L)	1.1 (0.4-3.1)	1.0 (0.5-2.4)	1.2 (0.4-3.1)	1.1 (0.5-2.8)	1.0 (0.5-2.8)	1.2 (0.5-3.5)
Neutrophils (×10^9^/L)	4.0 (3.1-4.7)	4.1 (3.3-5.1)	3.5 (2.6-4.5)	3.9 (3.1-4.7)	4.0 (3.3-4.7)	3.4 (2.9-4.3)
Smoking, n (%)	26 (35.6)	25 (65.8)	1 (2.9)	86 (45.7)	85 (64.9)	1 (1.8)
CAD family history, n (%)	33 (45.2)	19 (50.0)	14 (40.0)	85 (45.2)	57 (43.5)	28 (49.1)
Hypoglycemic therapy, n (%)	11 (15.1)	6 (15.8)	5 (14.3)	51 (27.1)^a^	31 (23.7)	20 (35.1)^c^
Anti-hypertensive therapy, n (%)	50 (68.5)	30 (78.9)	20 (57.1)	128 (68.1)	89 (67.9)	39 (68.4)
Lipid-lowering therapy, n (%)	11 (15.1)	6 (15.8)	5 (14.3)	62 (33.0)^aa^	43 (32.8)^b^	19 (33.3)^c^

CSI: Coronary stenosis index; BMI: Body mass index; W: Waist circumference; SBP: Systolic blood pressure; DBP: Diastolic blood pressure; FPG: Fasting plasma glucose; 2hPG: 2 h postprandial glucose; HbA1c: Glycated hemoglobin A1c; HOMA-IR: Homeostasis model assessment index; TC: Total cholesterol; TG: Triglyceride; UA: Uric acid; Scr: Serum creatinine; 24hALB: 24 h urine albumin; eGFR: Estimated glomerular filtration rate; CRP: C-reactive protein; Neutrophils: number of neutrophils.

For the total subjects: ^a^*p* < 0.05, CAD vs. non-CAD; ^aa^*p* < 0.01, CAD vs. non-CAD; for the men subgroup: ^b^*p* < 0.05, CAD vs. non-CAD, ^bb^*p* < 0.01, CAD vs. non-CAD; for the women subgroup: ^c^*p* < 0.05, CAD vs. non-CAD, ^cc^*p* < 0.01, CAD vs. non-CAD. Data are presented as mean ± SD or median (inter-quartile range).

### Relationship between serum LCN2 levels and CAD

**Figure 1:**
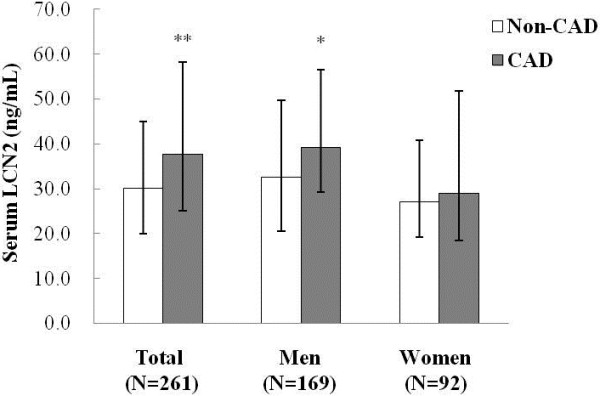
**Comparison of serum LCN2 levels between subjects with and without CAD.** White bars, non-CAD subgroup; grey bars, CAD subgroup. ** *p* < 0.01, CAD vs. non-CAD in total; * *p* < 0.05, CAD vs. non-CAD in men. Data are presented as median (inter-quartile range).

In general, the patients with CAD had significantly higher serum LCN2 levels than non-CAD subjects (37.6 (25.2-56.3) vs. 31.1 (19.9-45.0) ng/mL, *p* < 0.01). Men with CAD had significantly higher serum LCN2 levels than their counterparts without CAD (39.2 (29.3-56.5) vs. 32.7 (20.5-49.7) ng/mL, *p* < 0.05); however, the CAD-related significant trend was not found among the women (29.0 (18.5-51.9) vs. 27.0 (19.2-40.9) ng/mL, *p* > 0.05, Figure 1).

The gender-related significant findings prompted a focus of the multiple logistic regression analysis to identify factors that were independently correlated with CAD to be performed for the men cohort only. When CAD was set as the dependent variable, and age, BMI, HOMA-IR, LDL-c, serum adiponectin, eGFR, CRP, number of neutrophils, serum LCN2, smoking, CAD family history, hypoglycemic therapy, anti-hypertensive therapy, lipid-lowering therapy, MS and its components (central obesity, hypertension, hyperglycemia, hypertriglyceridemia, and low HDL-c) were set as the independent variables , serum LCN2 levels (odds ratio (OR) = 2.218, 95% confidence interval (CI): 1.017-4.839) and lipid-lowering therapy (OR = 3.428, 95% CI: 1.192-9.864) were identified as independent risk factors for CAD, while anti-hypertensive (OR = 0.360, 95% CI: 0.138-0.938) therapy was identified as an independent protective factor for CAD (all *p* < 0.05, Table 2).

**Table 2:** Multivariate logistic regression analysis showing factors independently associated with CAD in men

**Independent variable**	**β**	**S.E.**	***p***	**OR**	**95% CI**
Serum LCN2^@^	0.797	0.398	0.045	2.218	1.017-4.839
Anti-hypertensive therapy	-1.022	0.489	0.036	0.360	0.138-0.938
Lipid-lowering therapy	1.232	0.539	0.022	3.428	1.192-9.864

Variables of the original model included: age, BMI, HOMA-IR, LDL-c, serum adiponectin, eGFR, CRP, Neutrophils, serum LCN2, smoking, CAD family history, hypoglycemic therapy, anti-hypertensive therapy, lipid-lowering therapy, MS, central obesity, hypertension, hyperglycemia, hypertriglyceridemia, and low HDL-c.

^@^Data were natural logarithm transformed before analysis and only significant variables were presented.

### Relationship between serum LCN2 levels and MS in men

**Figure 2:**
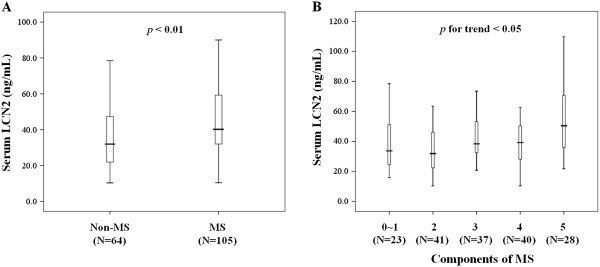
**Relationship between serum LCN2 levels and MS in men. (A)** Serum LCN2 levels in men with and without MS; **(B)***p* < 0.05 for trend of increased serum LCN2 levels in men with increasing numbers of MS components. The bars represent the median, and 25th and 75th percentile of serum LCN2 levels, respectively.

Similar to the CAD findings, men with MS showed significantly higher serum LCN2 levels than their non-MS counterparts (40.2 (31.9-59.4) vs. 32.0 (21.7-47.6) ng/mL, *p* < 0.01, Figure 2A). To further evaluate this relationship, the contribution of the extent of MS was evaluated by stratification analysis according to the number of MS components, using the following sub-groups: 0 ~ 1 component, n = 23; 2 components, n = 41; 3 components, n = 37; 4 components, n = 40; and 5 components, n = 28. The analysis showed a positive trend between increasing serum LCN2 levels and increasing number of MS components (*p* for trend <0.05, Figure 2B).

### Influencing factors of increased serum LCN2 levels

Both the men and women groups were examined to evaluate the correlation between clinical indicators and serum LCN2 levels. In men, serum LCN2 levels were found to be positively correlated with BMI (*r* = 0.195, *p* = 0.011), W (*r* = 0.164, *p* = 0.033), TG (*r* = 0.215, *p* = 0.005), CRP (*r* = 0.234, *p* = 0.003) and neutrophils (*r* = 0.166, *p* = 0.032). In women, CRP (*r* = 0.254, *p* = 0.017) and neutrophils (*r* = 0.249, *p* = 0.017) were correlated (all *p* < 0.05).

To further assess the independently correlated clinical parameters of serum LCN2 levels, multivariate stepwise regression analysis was performed in both the men and women groups, respectively. When serum LCN2 levels were set as the dependent variable, and age, BMI, W, SBP, DBP, FPG, 2hPG, HbA1c, HOMA-IR, TC, TG, HDL-c, LDL-c, serum adiponectin, UA, Scr, eGFR, CRP, number of neutrophils, hypoglycemic therapy, anti-hypertensive therapy and lipid-lowering therapy were set as the independent variables, TG (Standard β = 0.238, *p* = 0.003) and neutrophils (Standard β = 0.173, *p* = 0.031) were found to be independent factors of serum LCN2 levels in men while only neutrophils (Standard β = 0.286, *p* = 0.009) remained significant in women (all *p* < 0.05, Table 3).

**Table 3:** Multivariate stepwise regression analysis of serum LCN2 levels

**Independent variable**	**β**	**S.E**	**Standard β**	***p***
Men (N = 169)				
TG^@^	0.225	0.075	0.238	0.003
Neutrophils^@^	0.327	0.150	0.173	0.031
Women (N = 92)				
Neutrophils^@^	0.537	0.200	0.286	0.009

Variables of the original model included: age, BMI, W, SBP, DBP, FPG, 2hPG, HbA1c, HOMA-IR, TC, TG, HDL-c, LDL-c, serum adiponectin, UA, Scr, eGFR, CRP, Neutrophils, hypoglycemic therapy, anti-hypertensive therapy, and lipid-lowering therapy.

^@^Data were natural logarithm transformed before analysis and only significant variables were presented.

## Discussion

Lcn2 expression is closely related to metabolism and inflammation, both of which contribute to the pathogenic process of atherosclerosis. In addition, serum LCN2 levels are positively associated with the subsequent development of CAD in patients with coronary artery atherosclerosis. Furthermore, this relationship has been described in CAD patients of Caucasian and Asian (Korean) ethnic origin, with the former showing the positive association between CAD severity in patients with myocardial infarction (vs. those with stable angina) [16-18]. Increased serum LCN2 levels were also shown to be gender- (in men vs. women) and weight- (in obese vs. normal weight) related [5]. In the present study, obvious gender-related differences were observed as well (in agreement with the previous studies) which led to the study design including gender stratification analysis (with a men focus in the regression analysis). Ultimately, the Chinese men in our study who had CAD also had significantly higher serum LCN2 levels than either the women or their non-CAD men counterparts.

Studies to elucidate the pathogenic molecular mechanism of Lcn2 in atherosclerosis and CAD have shown that the Lcn2/MMP-9 complex acts to destabilize the artery plaque [25]. This complex has been detected in clinical specimens of plaques on the side facing the lumen area and in lipid centers, suggesting an involvement in vascular inflammation and reconstruction in myocardial ischemia [26]. MMP-9 alone, however, has been suggested to promote atherosclerosis by degrading the vascular basement membrane, thereby increasing endothelial permeability and allowing more white blood cells and inflammatory cytokines to infiltrate the intima. Indeed, the conditions of plaque bleeding and hypoxia are accompanied by increased levels of the Lcn2/MMP-9 complex; since the complexed form also acts to protect the MMP-9 protein from degradation, it is more capable of exerting a further effect (both in its beneficial and detrimental manners) on the surrounding tissue. The differential effects of MMP-9 have been seen in different stages of CAD. It is believed that the uncomplexed protein’s degradation might be initially beneficial to vascular repair. However, the constant inflammatory stimulation and up-regulated Lcn2 may lead to increased levels of the more stable Lcn2/MMP-9 complex, thereby destabilizing the plaques and prompting the acute cardiovascular event [27].

The current study included a cohort of patients at high risk of metabolic diseases, especially MS. The results suggested that serum LCN2 levels were 1.25-times higher in the patients with MS than those without MS; furthermore, the levels of LCN2 increased along with increases in the number of MS components in the men patients. Patients with metabolic disorders have been previously shown to have a higher rate of cardiovascular diseases [28]. MS, in particular, and its components (which are characterized as conventional risk factors of cardiovascular diseases) is known to cause endothelial damage leading to formation of the atherosclerotic plaque and facilitating lipid infiltration that contributes to atherosclerosis progression [29,30]. In agreement with this pathogenic process, serum HDL-c has been previously demonstrated as negatively correlated with serum LCN2 levels [18]; likewise, in the current study serum TG was identified as a positive influencing factor of serum LCN2 levels, indicating that the related pathogenic mechanism of atherosclerosis may involve disruption of lipid metabolism.

Chronic inflammation is another well-recognized feature of CAD development. Previous studies have shown that patients with acute coronary syndrome also have increased levels of serum CRP [31,32], which are positively associated with serum LCN2 levels even after adjusting for several confounders [5]. In this study, the number of neutrophils, generally known to be an index of inflammation status, was found to be positively correlated with serum LCN2 levels. Similar results were obtained in the current study of Chinese cohort, suggesting that LCN2 might prompt the inflammatory process that induces atherosclerosis.

### Limitations

When interpreting the results from the current study, three inherent limitations to the study design must be remembered. Firstly, the sample size was relatively small. Secondly, the cross-sectional nature of the study precluded the ability to confirm whether LCN2 itself was a predictor of CAD. Finally, additional factors that may influence in the outcome of our population were not measured, including MMP-9 [33], retinol binding protein 4 and resistin [34]. Subsequent investigations should consider each of these features to provide further insights into this pathogenesis topic.

## Conclusions

We conclude that the presence of increased serum LCN2 levels is closely associated with CAD and MS in a Chinese cohort. However, further large population-based prospective studies encompassing also other ethnicities are needed to demonstrate the potential of serum LCN2 as a sensitive predictor for CAD.

## Consent

Written informed consent was obtained from the patient for the publication of this report and any accompanying images.

## Abbreviations

BMI: Body mass index; CAD: Coronary artery disease; CAG: Coronary angiography; CRP: C-reactive protein; CSI: Coronary stenosis index; DBP: Diastolic blood pressure; eGFR: Estimated glomerular filtration rate; FPG: Fasting plasma glucose; HbA1c: Glycated hemoglobin A1c; HOMA-IR: Homeostasis model assessment index; LCN2: Lipocalin-2; MMP-9: Matrix metalloproteinases-9; MS: Metabolic syndrome; Neutrophils: Number of neutrophils; NGAL: Neutrophil-gelatinase associated lipocalin; SBP: Systolic blood pressure; Scr: Serum creatinine; TC: Total cholesterol; TG: Triglyceride; UA: Uric acid; W: Waist circumference; 2hPG: 2 h postprandial glucose; 24hALB: 24 h urine albumin.

## Competing interests

We declare that there is no conflict of interest that could be perceived as prejudicing the impartiality of the research reported.

## Authors’ contributions

YB and WJ designed the study. MZ, YH, JT and MG collected data. JN analyzed data and wrote the draft. XP measured serum LCN2. ZL did the angiographic analysis. XM, YB, and WJ revised the paper and contributed to the discussion. All authors read and approved the final manuscript.
